# Frequency of everyday pro-environmental behaviour is explained by baseline activation in lateral prefrontal cortex

**DOI:** 10.1038/s41598-018-36956-2

**Published:** 2019-01-09

**Authors:** Thomas Baumgartner, Benedikt P. Langenbach, Lorena R. R. Gianotti, René M. Müri, Daria Knoch

**Affiliations:** 1 University of Bern, Department of Social Psychology and Social Neuroscience, Institute of Psychology, Fabrikstrasse 8, University of Bern, 3012 Bern, Switzerland; 2 University Hospital Bern, Department of Neurology, University Neurorehabilitation, Freiburgstrasse 41c, 3012 Bern, Switzerland

## Abstract

Humankind faces a plethora of environmental problems, many of which are directly influenced by individual human behaviour. To better understand pro-environmental behaviour, we here try to identify interindividual markers that explain variance in the frequency of every-day pro-environmental behaviour. So far, research on this topic has mainly relied on subjective self-report measures and has yielded mixed results. In this study, we applied a neural trait approach to assess stable, objective individual differences. Using source-localised electroencephalography, we measured cortical activation at rest and combined our neural task-independent data with an ecologically valid assessment of everyday pro-environmental behaviour. We find whole-brain-corrected evidence that task-independent baseline activation in the right lateral prefrontal cortex, a brain area known to be involved in cognitive control and self-control processes, explains individual differences in pro-environmental behaviour. The higher the cortical baseline activation in this area, the higher the frequency of everyday pro-environmental behaviour. Implications for the promotion of pro-environmental behaviour are discussed.

## Introduction

It is well-established that immediate action is needed to avert a potentially catastrophic climate change^1,2^ and it has been argued that individual decision-making might be a promising target to reduce humankind’s negative influence on the environment^3–6^. However, while there is substantial interindividual variance in pro-environmental behaviour^7,8^, it is still not fully understood why some people exhibit pro-environmental behaviour while others do not, and how said behaviour can be fostered.

Traditional models employed in environmental psychology (like the theory of planned behaviour^9^ or the theory of reasoned action^10^, for an overview, see^11^) strongly focus on a person’s attitudes towards pro-environmental behaviours. It is therefore not surprising that the use of information campaigns to change attitudes and increase awareness has been a dominating approach to enhance pro-environmental behaviour. Recently however, criticism emerged from within the environmental science community^2,12^: Attitude change has turned out to be less effective than hoped^13^, and there is a mismatch between people’s pro-environmental attitudes and their pro-environmental behaviour. This “attitude-behaviour gap^14,15^” indicates the need to identify other factors explaining why some people behave sustainably while others do not. One possible explanation would be that there are strong environmental factors influencing behaviour. For example, sometimes there just is no infrastructure provided to recycle or to use public transport^16^. Additionally, and corresponding to traditional models like the norm activation theory^17^ and the focus theory of normative conduct^18^ (for an overview, see^11^), it has been shown that the behaviour and norms of other people in a person’s environment largely influence pro-environmental behaviour^19,20^.

But how can one reliably identify a “green person” (someone with a general proclivity for green behaviour across many situations)? Naturally, many researchers turned to traditional trait models like the Big Five^21^, typically assessed using self-reports. But while the relationship between broad personality traits and pro-environmental behaviour has sparked a considerable amount of research, no clear pattern as of which traits are related to pro-environmental behaviour has emerged, and findings are sometimes contradictory^22–24^.

In this study, we aimed to find a trait that explains pro-environmental behaviour using an objective measurement of individual differences. For this purpose, we applied the neural trait approach, which identifies task-independent, brain-based differences between people and links these differences to a behaviour of interest^25^. Specifically, we recorded task-independent resting electroencephalography (EEG) of 87 participants before measuring their everyday pro-environmental behaviour over the course of five days. Measuring an individual’s baseline cortical activation with EEG is an ideal neural trait measure because it is unique to an individual (recognising a person based on their pattern of brain electrical activity at rest is possible in up to 99% of cases) and relatively stable over time, with a retest-reliability of up to 0.80 after 5 years^26–28^. Prior studies could show that this measure of neural traits helps to explain variance in expertise (e.g. musicianship^29^), intelligence (e.g. psychometric intelligence^30^), and social and non-social behaviour, such as for example deception^31^, alcohol consumption^32^, cooperation^33^ and norm-compliance^34^.

Of course, the fact that we here focus on establishing a trait that explains differences in pro-environmental behaviour across different situations does not mean that situational factors can or indeed should be disregarded. Similarly, it is known that sociodemographic variables like income, level of education, or age also effect pro-environmental behaviour^35^. Still, exploring which traits influence inter-individual differences in pro-environmental behaviour is an equally important topic. To the best of our knowledge, this is the first study that uses a neural trait approach to explain interindividual differences in everyday pro-environmental behaviour. We therefore chose an exploratory analysis and applied a whole-brain-corrected approach (not restricting the analysis to any a priori hypothesised region).

To measure pro-environmental behaviour, we used experience-sampling^36^, which takes advantage of the wide-spread use of smartphones and is known to enhance ecological validity and reduce recall bias^37^. Typically, participants receive multiple short questionnaires a day to immediately fill out. Because they only have to remember actions from the last couple of hours and data is collected over multiple days, this method enables participants to accurately recall their behaviour^38^. Combining experience sampling with the neural trait approach, we aimed to establish an objective neural trait, which explains variance in pro-environmental behaviour. Additionally, participants reported their general environmental attitudes on the New Environmental Paradigm (NEP)^39^. This enabled us to compare the predictive power of the neural trait approach with an established measurement of environmental attitudes.

## Results

**Figure 1:**
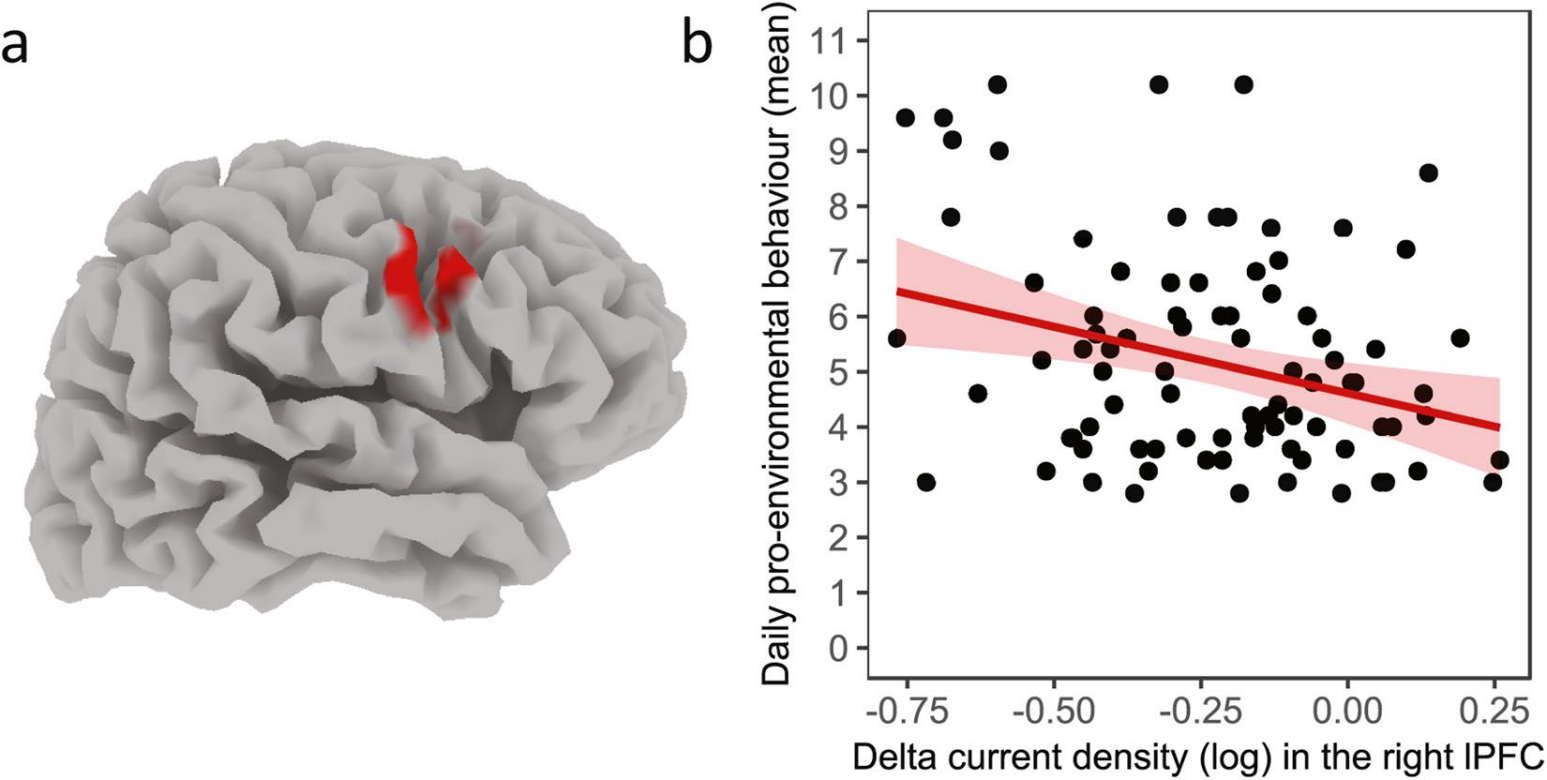
Depiction of the correlation between delta current density and pro-environmental behaviour. Panel a shows the area in the right lateral prefrontal cortex where baseline delta current density is statistically significantly correlated with pro-environmental behaviour (whole-brain corrected). Panel b shows a corresponding scatter plot with a robust regression line (which accounts for potential outliers) and 95%-confidence intervals. Depicted is the relationship between baseline delta current density in the right lateral PFC (10 mm sphere around the peak voxel) and pro-environmental behaviour. Baseline slow wave oscillations likely reflect decreased cortical activation, thereby suggesting that higher baseline activation in the right lateral PFC is related to more environmentally friendly behaviour.

On average, participants had a pro-environmental behaviour score of 5.29 (SD: 1.97; range: 2.80–10.20). On the NEP, they showed a mean of 3.35 (SD: 0.43; range: 2.37–4.36). The whole-brain-corrected analyses showed statistically significant negative correlations between current density in the right lateral prefrontal cortex [MNI-coordinates: x = 60; y = 10; z = 30, Brodmann area 6/9] in the delta band and pro-environmental behaviour (p < 0.05, whole-brain corrected). A robust regression (M estimation with iterated re-weighted least squares) using the mean current density in a ROI (10 mm sphere around the peak voxel) resulted in a correlation coefficient of −0.30, p = 0.004, R^2^ = 0.09 (see Fig. 1). Partialling out participants’ gender and age did not affect the relation between current density in the delta band and pro-environmental behaviour (r = −0.29, p = 0.006, R^2^ = 0.084). As baseline slow wave oscillations likely reflect decreased cortical activation^40–42^, our findings suggests that people with higher baseline activation in the right lateral PFC at rest behave in a more environmentally friendly way.

Additionally, we wanted to explore whether baseline activation in the right lateral PFC can explain unique variance which cannot be explained by general attitudes towards the environment. To this end, a hierarchical regression was conducted with pro-environmental behaviour scores as dependent variable, and NEP score and delta current density in the right lateral PFC as first and second predictor, respectively. Due to the nine participants who did not fill out the NEP, these analyses have been calculated with N = 78. The NEP score correlated with daily pro-environmental behaviour on a trend level (p = 0.09). Including the second predictor into the regression raised the explained variance from 3.8% to 13.4%, and an ANOVA proofed the difference between the two models to be statistically significant, F(1, 75) = 8.31, p = 0.005, Cohen’s f^2^ = 0.11 (corresponding to a small effect). Hence, baseline activation in the right lateral PFC does indeed explain unique variance.

## Discussion

Using an exploratory whole-brain neural trait approach, we report evidence that a specific neural trait – task-independent baseline activation in the right lateral prefrontal cortex – partly explains daily pro-environmental behaviour in the field. The higher the level of baseline activation in the lateral prefrontal cortex, the more pro-environmental behaviour was reported by the participants. We therefore propose that baseline activation in the right lateral PFC is a stable marker for every-day pro-environmental behaviour.

While one has to be careful with interpreting exploratory findings, our results fit well into the established literature on the role of the lateral PFC in decision-making. A substantial body of research shows that higher levels of both baseline and task-related activation in the lateral PFC correlates with increased self-regulation, inhibitory control, or executive functions in general^32,43–47^. Additionally, EEG baseline activation of the lateral PFC is also related to established task-related neural markers of self-control like the NoGo-Anteriorisation and the NoGo-P300^34,48^. Converging evidence comes from the study of a different neural trait approach, namely anatomical brain differences measured using structural magnetic resonance imaging (MRI), with evidence suggesting that structural MRI and resting EEG are correlated^49^. It has been shown that cortical thickness in the lateral PFC is positively correlated with the ability to suppress impulsive behaviour^50^, and as the lateral PFC matures, children are increasingly capable of suppressing egoistic impulses to act according to more complex long-term goals^51,52^. Finally, neuromodulation studies showed that inhibiting the lateral PFC diminishes the ability to excerpt self-control in social and non-social contexts^53–58^.

In the realm of pro-environmental behaviour, a similar process might be involved. It is well possible that the implementation of pro-environmental behaviour in everyday situations requires active self-control to not succumb to temptations^59^. If people’s self-control is low (corresponding to less activation in the lateral PFC), they might not be able to stick to their initial intentions (e.g., conserving water) in the light of tempting behavioural alternatives (e.g., taking a bath after a long day at work), even if they do care about the environment. This might also explain why general attitudes towards the environment are not as ideal a predictor of pro-environmental behaviour as one could expect, with pro-environmental behaviour being a typical case of a “willing spirit” meeting “weak flesh”. On the other hand, people with a high baseline activation in the lateral PFC might be better at considering their original intentions when presented with a tempting behavioural alternative.

Our finding fits well into recent theorising in environmental science. Indeed, multiple researchers have proposed that it might be crucial to take self-control processes into account when trying to encourage pro-environmental behaviour^2,12^, but empirical evidence for this proposal is rare^60^. Our results, however, grant neuroscientific support to the idea that self-control is involved in everyday pro-environmental decision-making.

Resting EEG (and resting fMRI) might also have some limitations. First, performing a resting state recording might not be as straightforward as it seems; participants’ experiences and stimulus independent thoughts may vary despite similar instructions^61^. Controlling these spontaneous thoughts is difficult, but consistent activation patterns of resting state networks among studies suggest only a limited influence^62^. Second, because the sLORETA solution space is restricted to the cortical grey matter and the hippocampus, future studies could apply other methods (e.g. structural MRI, resting functional MRI) to explore whether neural trait measures of subcortical areas can also be used to predict pro-environmental behaviour.

It is important to note that while our data provide evidence for a stable neuroscientific trait, “stable” does not equal “unchangeable”: Indeed, the human brain is known to change throughout adulthood^63^ and techniques like self-control trainings, neurofeedback, repeated practice or meditation can have lasting effects on neural structure and functioning of the prefrontal cortex^64–70^. Drawing on this knowledge, our research could help to find new ways to promote pro-environmental behaviour in those who so far struggle to behave sustainably.

## Methods

### Participants

Participants consisted of 87 healthy students from the University of Bern (66 female; mean age ± SD: 20.9 ± 2.0 years). All participants gave written informed consent; the study was approved by the local ethics committee of the Faculty of Human Sciences (University of Bern, Switzerland) and conducted in accordance to the declaration of Helsinki. Participants received a compensation of 25 Swiss Francs (CHF; 1 CHF ≈ 1 US dollar) and an additional 2 CHF for every time they completed the experience sampling, resulting in a maximum additional amount of 30 CHF.

### Procedure

Resting EEG data was acquired in the EEG laboratory, where only one participant was tested at a time. For the experience sampling part of the study, our participants were invited to the behavioural lab and registered with the online software through which they would receive the invitations to the experience sampling questions via text message. Then, they were familiarised with the use of the experience sampling method on their smartphones and the content of each item. The actual experience sampling started two days later. An online-version of the New Environmental Paradigm (NEP)^39^ was distributed to the participants after the experience sampling. We implemented a gap of several weeks between EEG-recording, experience sampling, and assessment of the NEP to minimise any potential carry-over effect.

### Recording and processing of EEG data

The EEG acquisition took place in a sound and electrically shielded chamber that was dimly lit and contained an intercom connection to the experimenters. We used BrainVision Recorder (version 1.20) to record the EEG during rest with open or closed eyes; the instructions about eye opening/closing were given via intercom. The protocol consisted of 20-s eyes open followed by 40-s eyes closed, repeated 5 times (such a protocol guarantees minimal fluctuations in participants’ vigilance state). Data analysis is based on the 200-seconds eyes-closed condition. We recorded a continuous EEG from 60 Ag/AgCl electrodes placed according to the international 10–10 system^71^, using a BrainAmp DC amplifier with a sampling rate of 500 Hz (bandwidth: 0.1 to 250 Hz). The electrode at the position FCz was used as recording reference and the electrode at the position CPz as ground electrode. Horizontal and vertical electro-oculographic signals were recorded with two additional electrodes at the left and right outer canthi of the left and right eye and an additional electrode below the right eye. Impedances were kept below 10 kΩ. BrainVision Analyzer (version 2.1.2) was used for preprocessing the EEG data. We filtered the EEG data offline, using a high pass filter of 0.5 Hz and a low pass filter of 30 Hz, notch filter enabled at 50 Hz. EEG signals with excessive noise were replaced using a spline interpolation of the signal of adjacent electrodes. To remove eye movements artefacts, we first ran an Independent Component Analysis (ICA) and then manually removed factors related to horizontal and vertical eye movements (usually only two factors were detected and eliminated). After having removed the factors related to eye movements, the EEG was recomputed using an inverse ICA procedure. Then we applied an automatic artefact rejection with the following parameters: maximal voltage step: 15 μV; maximal amplitude: ±100 μV; minimal allowed activity in intervals of 100-ms length: 0.5 μV. After this automatic artefact rejection, data were also visually examined to eliminate residual artefacts. Further, data were recomputed against the average reference. All artefact-free 2-s EEG-epochs were extracted. On average, there were 88 (SD: 20.5) epochs per person. A Fast Fourier Transformation (using a square window) was applied to each epoch and channel to compute the power spectra with 0.5-Hz resolution. The spectra for each channel were averaged over all epochs for each participant. Absolute power values were obtained for the following seven independent frequency bands^72^: delta (1.5–6 Hz), theta (6.5–8 Hz), alpha1 (8.5–10 Hz), alpha2 (10.5–12 Hz), beta1 (12.5–18 Hz), beta2 (18.5–21 Hz), and beta3 (21.5–30 Hz). The intracortical electrical sources that generated the scalp-recorded activity in each of the seven frequency bands were estimated using the standardised low-resolution brain electromagnetic tomography (sLORETA, version v20160611)^73^. The sLORETA method is a properly standardised discrete, 3D distributed, linear, minimum norm inverse solution. The particular form of standardisation used in sLORETA endows the tomography with the property of exact localisation to test point sources, yielding images of standardised current density with exact localisation, albeit with low spatial resolution (i.e., neighbouring neural sources will be highly correlated). sLORETA has been validated in several simultaneous EEG/fMRI studies^74,75^ and in an EEG localisation study for epilepsy^76^. In the current implementation of sLORETA, computations were made in a realistic head model using the MNI152 template^77^, with the 3D solution space restricted to cortical grey matter, as determined by the probabilistic Talairach atlas^78^. The intracerebral volume is partitioned in 6239 voxels at 5 mm spatial resolution. Thus, sLORETA images represent the standardised electric activity at each voxel in neuroanatomic Montreal Neurological Institute (MNI) space as the exact magnitude of the estimated current density (unit: amperes per square meter, A/m^2^). Using the automatic regularisation method in the sLORETA software, we chose the transformation matrix with the signal-to-noise ratio set to 10. In order to reduce confounds that have no regional specificity, such as inter-subject variability in total power, a global normalisation and log-transformation of the sLORETA images was carried out prior to subsequent statistical analyses.

### Assessment of daily pro-environmental behaviour

For five consecutive days, participants received the link to the daily pro-environmental questions three times daily (late morning, afternoon, evening). For this, they received a link via text message (using the SurveySignal online service). The online questions on daily pro-environmental behaviour were implemented using the Qualtrics survey software. After the experience sampling was completed, participants received information about their final compensation and were paid. Every set of experience sampling consisted of five questions. Rather than focusing on one specific aspect, we assessed a wide range of everyday pro-environmental behaviours (not littering in the street; separating waste; not buying products that are not environmentally friendly; paying attention to using little water; ordering coffee in a reusable cup rather than a paper cup). Participants were asked to indicate whether they had shown these behaviours since the last time they had answered the questions. The assessment of daily pro-environmental behaviour took place during the semester, so that that participants (all of whom were students) had plenty of opportunities to experience situations in which they could behave pro-environmentally, and so that all participants had a fairly similar daily routine. Additionally, we asked to report whether there were any extraordinary circumstances that prevented them from showing the behaviours in questions (e.g., whether they were sick at home). This did not occur.

On average, participants responded at 14.8 of the 15 experience sampling time points (SD: 0.7; range: 12–15). To analyse participants’ pro-environmental behaviour, we first calculated the sum of pro-environmental behaviour per day and then computed the arithmetic mean over all 5 days, so that participants could have a pro-environmental behaviour score between 0 and 15.

### General attitudes towards the environment

In order to examine whether individual differences in cortical baseline activation are capable of explaining unique variance in pro-environmental behaviour compared to an environmental attitude measure, we also employed the New Environmental Paradigm^39^, the most widely used measure of environmental concern^4,79^. It consists of 15 items about environmental views (e.g., “If things continue on their present course, we will soon experience a major ecological catastrophe” or “Humans are seriously abusing the environment”). The NEP has an alpha of 0.83^39^. Dunlap *et al*.^39^ also provide a good overview over the relevant literature regarding the NEP’s validity. For example, the NEP has been shown to correlate with behavioural intentions and behaviours. Additionally, groups who would theoretically be expected to hold more pro-environmental attitudes (e.g., members of environmental groups) indeed score higher on the NEP. The content validity has been confirmed by ethnographic interviews, lending support from qualitative research as well. Participants responded on a 5-point Likert scale, and we used the mean over all items (reverse coding the anti-NEP items, so that higher scores correspond to more pro-environmental attitudes) for further analyses, resulting in a pro-environmental score between 1 and 5. Out of the 87 participants, 78 completed the NEP.

### Statistical analysis

The main goal of this study was to examine whether pro-environmental behaviour can be explained based on a task-independent neural trait. For this purpose, we decided to run robust regression analyses to minimize the influence of potential outliers. Since robust regressions are not implemented in the sLORETA software, normalised and log-transformed current density values for each voxel, frequency band, and participant were exported from sLORETA to Matlab. We then used a custom made script that allows to run robust regression analyses in the whole-brain (sLORETA solution space; 6239 voxels) and to conduct non-parametric statistics in order to incorporate correction for multiple comparisons^80^. This approach uses a randomisation strategy that determines the values of the critical probability threshold (at p < 0.05, corrected) for the observed r-values to identify cortical voxels that significantly correlate with the measures of interest in each frequency band.

## Data Availability

The datasets generated and analysed during the current study are available from the corresponding author on reasonable request.
